# Of mice, macaques and men: scaling of virus dynamics and immune responses

**DOI:** 10.3389/fmicb.2015.00355

**Published:** 2015-04-23

**Authors:** Christian L. Althaus

**Affiliations:** Institute of Social and Preventive Medicine (ISPM), Faculty of Medicine, University of BernBern, Switzerland

**Keywords:** allometric scaling, viral dynamics, viral replication, immune escape, cellular immune response, mice, macaques, humans

In this Opinion piece, I argue that the dynamics of viruses and the cellular immune response depend on the body size of the host. I use allometric scaling theory to interpret observed quantitative differences in the infection dynamics of lymphocytic choriomeningitis virus (LCMV) in mice (*Mus musculus*), simian immunodeficiency virus (SIV) in rhesus macaques (*Macaca mulatta*) and human immunodeficiency virus (HIV) in humans.

It is well-known that the average metabolic rate of cells is typically lower in larger species (Kleiber, 1932). Allometric theory predicts that the total metabolic rate of an organism scales approximately as *M*^3/4^, with *M* being the body mass (West et al., 1997). Assuming invariant size and volume of cells between species, the metabolic rate of a single cell then scales as *M*^−1/4^. Other properties such as the lifespan of an animal or the number of certain cell types have also been found to depend on body size (Peters, 1983; Schmidt-Nielsen, 1984; Calder, 1996; Savage et al., 2007). In host-pathogen systems, the following three aspects could underlie allometric scaling laws (Wiegel and Perelson, 2004; Cable et al., 2007; Banerjee and Moses, 2010). First, the metabolic rate will affect the rate at which cells synthesize DNA and proteins and could therefore influence the replication rate of viruses. Second, quantitative processes of the cellular immune response could be affected by allometric scaling. Third, the interaction between viral replication rates and the dynamics of immune responses could result in differences in the time to disease progression between host species.

Insights of the within-host dynamics of HIV is often based on experimental studies in macaques. Despite being a non-natural host, macaques can be infected with SIV, the simian counterpart of HIV. The viral turnover within a host has been found to be more rapid in macaques than in humans, with estimated half-lives of virus-producing cells of 0.5 days and 0.7 days, respectively (Markowitz et al., 2003; Brandin et al., 2006). In this light, it is interesting to compare the dynamics at which immune escape variants evade recognition from CD8^+^ cytotoxic T lymphocyte (CTL) responses in the two host species. Asquith and McLean (2007) showed that escape rates in macaques are about twice as fast as in humans, and suggested more efficient CTL-mediated killing of infected cells in macaques. An alternative hypothesis would be that the rate of immune escape depends on the overall viral turnover (Althaus and De Boer, 2012). With a 10-fold difference in body mass between humans (~70 kg) and macaques (~7 kg), allometric scaling predicts a 1.8-fold difference in the metabolic rate of a single cell between the two species. This factor is close to the reported 2-fold difference in escape rates and the 1.4-fold difference in the viral turnover between humans and macaques. This suggests that the metabolic rate could indeed directly affect the rate of virus replication and indirectly influence the rate of immune escape. However, it is worth noting that rates of CTL escape show substantial variation within host species and are generally faster during the acute phase of infection compared to the chronic phase (O'Connor et al., 2002; Asquith et al., 2006; Althaus and De Boer, 2008).

Previous studies have investigated how quantitative processes of the cellular immune response could be affected by allometric scaling. In the case of T cell responses, Wiegel and Perelson (2004) derived some general principles on how the number of naive T cells scales with body size. Lymphocyte trafficking, i.e., the circulation of T cells through blood, tissues and the lymphatic system in order to recognize antigen and eliminate virus-infected cells, has also been suggested to underlie general scaling laws (Perelson and Wiegel, 2009). Scaling principles could also be reflected in the varying life spans of naive and memory T cells between mice and men (De Boer and Perelson, 2013). In addition, the proliferation rate of CD8^+^ T cell responses during the acute phase of an infection could be linked to the metabolic rate of the host. CD8^+^ T cells have been found to expand rapidly with a rate of around 2.0 per day after LCMV infection in mice (De Boer et al., 2001, 2003; Althaus et al., 2007). The CD8^+^ T cell kinetics in those studies was measured for acute LCMV infection with the Armstrong strain. Other clones or infection models can cause persistent infection (Recher et al., 2007; Mueller et al., 2007), but the proliferation rates of CD8^+^ T cells during the acute phase of infection appear to be similar (Althaus et al., 2007). The expansion rate of CD8^+^ T cell responses upon SIV-infection in macaques is only about 1.0 per day (Davenport et al., 2004) and suggests a two-fold difference in proliferation rates between the two species. Allometric theory would predict a 4.3-fold difference between the metabolic rates of a single cell in mice (~20 g) and macaques (~7 kg). Hence, differences in the metabolic rate alone cannot account for the different proliferation rates of CD8^+^ T cell responses between the two species.

The time to acquired immunodeficiency syndrome (AIDS) during HIV and SIV infection can be used as a quantity for disease progression. The wide variation of this rate in HIV-infected humans, ranging from years to decades (Fraser et al., 2007), makes it difficult to compare it to the more rapid disease progression of 1 to 2 years in SIV-infected macaques (Kestler et al., 1990). Cable et al. (2007) found that disease progression to symptoms and to death in a small set of pathogens in different host species scales with *M*^−1/4^. It seems unlikely that progression to AIDS during HIV and SIV infection scales according to this law, as that would suggest a 1.8-fold difference only. If disease progression were directly proportional to body mass, i.e., isometric, we would expect a 10-fold difference which seems to be more consistent with observed progression rates in humans and macaques. It is important to note that host genetics can play a significant role in suppressing SIV replication and establishing a persistent infection (Kirmaier et al., 2010), and is strongly associated with disease progression in HIV (Fellay et al., 2007). An independent marker of disease progression in HIV and SIV is the set-point viral load (Watson et al., 1997), which might be influenced by the viral replication rate. Thus, it remains open whether there is an allometric relationship for the time to disease progression during HIV and SIV infection, and what exponent it would have.

In summary, there are indications that viral replication and the proliferation of CD8^+^ T cell responses are slower in larger animals. Whether this influences the ability of the cellular immune responses to eradicate viruses during the acute phase of an infection remains unclear. Others have argued that the response rate of immune systems does not change systematically with body size. Instead, the sub-modular architecture of the immune system, where the number and size of lymph nodes increase sublinearly with body size, could balance the tradeoff between the local detection of pathogens and the global host response (Banerjee and Moses, 2010).

Discrepancies between experimental findings of the mice and human immune system have been described and illustrate that using mice as preclinical models for the study of human diseases can be challenging (Mestas and Hughes, 2004). Besides those differences that could be a result of separate evolution, I have highlighted some apparent quantitative differences between the dynamics of viruses and immune responses in mice, macaques and men. Those suggest that allometric scaling principles should be considered for the interpretation of observed differences in the infection dynamics between host species. Precise quantitative measurements of virus and immune response dynamics in different host species are scarce. This poses a problem for investigating scaling relationship in host-pathogen systems that should be addressed by both experimentalists and theoretical biologists. Only additional data on the kinetics of virus replication and the dynamics of T cell responses in different host species will allow to shed more light on the question whether the nature of viral infections is affected by the body size of their hosts.

## Conflict of interest statement

The author declares that the research was conducted in the absence of any commercial or financial relationships that could be construed as a potential conflict of interest.
